# Probably the *Mycobacterium avium* Complex around the Staple Line Exacerbated after Radiation Therapy

**DOI:** 10.1155/2022/9000493

**Published:** 2022-01-07

**Authors:** Masashi Ito, Akihiro Yoshii, Takashi Osaki, Tomohito Kuwako, Ei Yamaki, Osamu Kawashima

**Affiliations:** ^1^Department of Allergy and Respiratory Medicine, Gunma University Hospital, 3-39-15 Showa-machi, Maebashi 371-8511, Gunma, Japan; ^2^Department of Respiratory Medicine, Shibukawa Medical Center, 383, Shiroi, Shibukawa 377-0280, Gunma, Japan; ^3^Department of Thoracic Surgery, Shibukawa Medical Center, 383, Shiroi, Shibukawa 377-0280, Gunma, Japan

## Abstract

The space around the staple line after lung surgery is at high risk of nontuberculosis *Mycobacterium* pulmonary disease (NTM-PD). Solitary nodules of NTM-PD around the staple line are difficult to distinguish from lung cancer. There is no clear identification from laboratory data and radiologic findings without histological examination. In the present case, we misdiagnosed the pulmonary granulomas with *Mycobacterium avium* complex pulmonary disease (MAC-PD) as a recurrence of lung cancer. We conducted radiation therapy. The pulmonary granulomas with MAC-PD were exacerbated by irradiation. The effects of radiation therapy for MAC-PD are unknown. When radiation therapy is performed for the patient coexistence with MAC-PD, we should pay attention to exacerbation of MAC-PD.

## 1. Introduction

The annual prevalence of nontuberculous mycobacterial pulmonary disease (NTM-PD) has been increasing worldwide [1]. The *Mycobacterium avium* complex (MAC) is the most common bacterial species causing NTM-PD.

Several studies have reported pulmonary granulomas in recent years due to NTM-PD around the staple line after lung segmentectomy [2, 3]. However, solitary nodules of NTM-PD are difficult to distinguish from lung cancer without performing surgical lung resection and subsequent histological examinations. Herein, we report the misdiagnosis of a solitary nodule of MAC pulmonary disease (MAC-PD) around the staple line as recurrent lung cancer, which was incorrectly treated using radiation therapy.

Although studies have reported the worsening of tuberculosis due to radiation therapy [4–6], the relationship between the exacerbation of NTM-PD and radiation therapy remains uncertain. In this case report, we describe the possibility of exacerbation of MAC-PD after radiation therapy. Written consent was obtained from the patient to present this case.

## 2. Case Presentation

**Figure 1:**
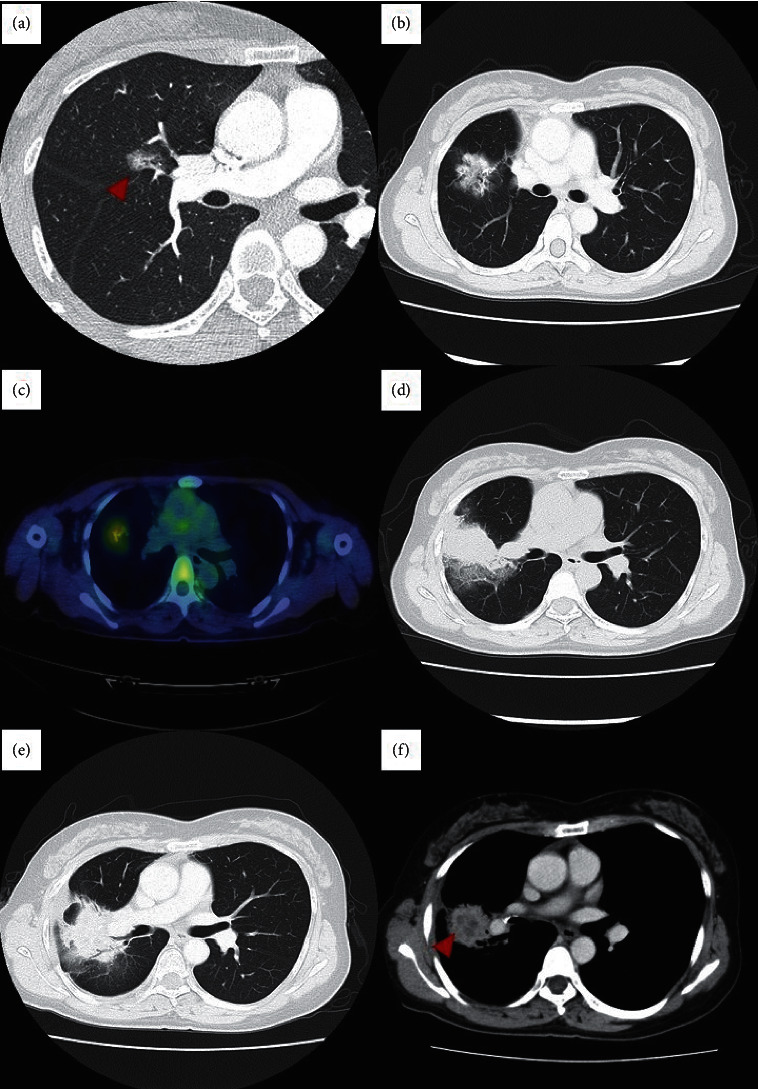
Chest computed tomography images. (a) A part-solid GGN on the middle lobe of the right lung. This portion of the GGN invades the upper lobe. (b) Three years and two months after the lung surgery, a mass emerges adjacent to the staple line. (c) Fluorodeoxyglucose PET-CT. The maximum standardized uptake value is 2.64. (d) Two months after the end of radiation therapy, ground-glass attenuation surrounding the mass and an increase in mass size are detected. (e) Five months after the end of radiation therapy, the mass shows continuous growth. (f) Five months after the end of radiation therapy, contrast-enhanced CT showed internal low absorption suspected of necrosis.

A 50-year-old woman presented with an abnormal chest shadow. Chest computed tomography (CT) revealed a part-solid ground-glass nodule (GGN) in the middle lobe of the right lung; moreover, a portion of the tumor had invaded the upper lobe (Figure 1(a)). The maximum diameter of the part-solid GGN was 15 mm. It remained after a month. She was a nonsmoker patient. She had a medical history of Sjögren's syndrome but was not on medication. She had no history of respiratory diseases including bronchiectasis. Because primary lung cancer could not be denied, lung lobectomy of the right middle lobe and partial lung resection of the right upper lobe (S3) were performed. Pathological findings confirmed the diagnosis of adenocarcinoma, pT2a(pl1)N0M0, pStage IA. The patient was administered uracil-tegafur at 400 mg/day for 2 years. After the surgery, we performed follow-up CTs regularly. It had passed without any significant change on CTs.

Three years and two months later, chest CT revealed a mass adjacent to the staple line (Figure 1(b)). The maximum standardized uptake value (SUVmax) measured using fluorodeoxyglucose positron emission tomography/CT (PET-CT) was 2.64 (Figure 1(c)). No lesions other than the lung masses were observed. The mass was located between the lobes, and it was difficult to perform a biopsy by surgical lung resection. Other biopsies were also judged to be difficult at this point due to the morphology of the lesion. Because the mass was suspected to be a local recurrence of lung adenocarcinoma, radiation therapy was administered (66 Gy/33 fr). Two months after the end of radiation therapy, ground-glass attenuation surrounding the mass and an increase in mass size were detected (Figure 1(d)). Because the patient was asymptomatic, radiation pneumonitis was suspected. An assessment conducted five months after radiotherapy revealed that the mass was continuously growing (Figure 1(e)). Contrast-enhanced CT showed internal low absorption suspected of necrosis (Figure 1(f)). Therefore, diseases other than radiation pneumonitis which include lung cancer and infectious diseases were suspected, and bronchoscopy was performed.

**Figure 2:**
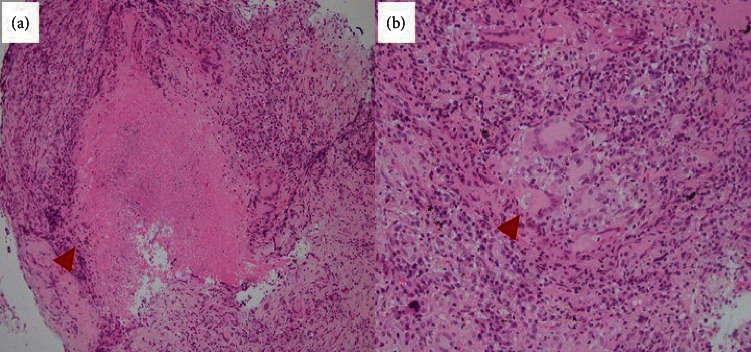
Histological findings revealed epithelioid granulomas with caseous necrosis and infiltration of lymphocytes with hematoxylin and eosin staining. (a) ×100 manifestation; (b) ×200 manifestation.

Histological examination of the transbronchial lung biopsy specimens revealed epithelioid granulomas with caseous necrosis and lymphocyte infiltration (Figure 2). No malignant cells were found, and Ziehl–Neelsen staining for acid-fast bacilli was negative. Although antiglycopeptidolipid (GPL) core IgA antibody for MAC and interferon-gamma release assays were negative, a culture test for *M. avium* in the bronchial lavage fluid (BAL) was positive. Accordingly, the mass adjacent to the staple line was identified as MAC-PD from the diagnosis criteria in recent guideline [7]. Because the patient was asymptomatic and, subsequently, radiological progression had stopped, treatment for MAC-PD was not administered.

## 3. Discussion

In the present case, we misdiagnosed pulmonary granulomas due to *Mycobacterium avium* complex pulmonary disease (MAC-PD) as a recurrence of lung cancer. Distinguishing solitary nodules from lung cancer requires histological diagnosis through percutaneous needle aspiration biopsy, transbronchial lung biopsy, or lung surgery. However, the mass was difficult to perform a biopsy in the present case. Previous studies have reported cases of pulmonary granulomas due to MAC-PD around the staple line after lung surgery [2, 3]. Solitary nodules of NTM-PD, regardless of the staple line, are also difficult to distinguish from lung cancer. The frequency of NTM-PD mimicking lung cancer is 3.6% [8], and PET-CT is ineffective in its diagnosis [9]. The median SUVmax of solitary nodules of NTM-PD has been reported to be 2.7–4.9 [8, 10]. CT features of NTM-PD nodules include poor contrast enhancement and internal calcification [8].

In the present case, ground-glass attenuation surrounding the mass and an increase in mass size were detected after radiation therapy. This radiological change reflected radiation pneumonitis and exacerbation of MAC-PD by radiation therapy. The effects of radiation therapy on MAC-PD remain unknown. Only one previous study reported a case of exacerbation of MAC-PD caused by irradiation [11]. Although some case reports have documented the occurrence or reactivation of pulmonary *Mycobacterium tuberculosis* due to radiation therapy [4–6], little is known about the mechanisms of *M. tuberculosis* reactivation by radiation therapy. Changes in immune cell balance, including lymphocytes, caused by radiation therapy are suspected to be involved in the reactivation of *M. tuberculosis*.

The limitations in this case report were the lack of tissue culture and treatment for MAC-PD, and the possibility of deterioration of MAC-PD in its natural course could not be denied. There was also the possibility of contamination of *M. avium* in BAL culture. It is difficult to conclude that MAC-PD was exacerbated by radiation in this case, but it is possible.

In conclusion, when nodules are detected around the staple line, benign granuloma due to MAC-PD should be suspected, and the diagnosis should be confirmed by histological examination. When radiation therapy is administered to patients with coexisting MAC-PD, attention should be paid to the exacerbation of MAC-PD.

## Data Availability

The case report data used to support the findings of this study are included within the article.
